# Resource Review: EndNote 21 desktop version

**DOI:** 10.5195/jmla.2023.1803

**Published:** 2023-10-02

**Authors:** Terri Gotschall

**Affiliations:** 1 terri.gotschall@ucf.edu, Scholarly Communications Librarian, Health Sciences Library, University of Central Florida College of Medicine, Orlando, FL.

## Abstract

**EndNote 21 desktop version.** Released May 2023. Clarivate, 1500 Spring Garden Street, Fourth Floor, Philadelphia, PA 19130; https://endnote.com/; 1-888-418-1937; onetime purchase full license, $274.95, discounts available, contact for institutional pricing. For a list of technical requirements, visit https://endnote.com/product-details/compatibility.

This review is an update of the Resource Review for EndNote 20 which included technical requirements, comparison to other products, and an overview of the user interface. As EndNote 21 operates differently on a Mac this review focuses on personal computers. EndNote 21 was released in May of 2023 and has some notable upgrades from the EndNote 20 version. EndNote, Clarivate's reference management software, allows users to import, organize and maintain bibliographic records. EndNote 21 has the same decluttered user interface as EndNote 20, and the essentials like importing records, adding records manually, and citing references in Word have not changed.

The most notable change in the desktop version is the addition of Tags. Users can now create custom color tags and apply them to individual records. For those with color accessibility needs, prior to renaming the tag, the name of the color is written out beside the tag. However, the addition of a symbol or number would make the tags more accessible when viewing them in the library. Tagging adds an additional way to organize content quickly and easily. To add a tag, click on the record. At the top of the record click on “Manage Tags.” In the popup window, a user can add a tag, delete a tag, or create a new tag. Once one or more tags are created, the heading “Tags” appears in the left-hand sidebar. Similar to “Groups,” clicking on the tag in the sidebar shows all of the records with that tag.

Another upgrade to EndNote 21 is that users can now share a synced library with up to 1,000 users. This is a significant increase from being able to share with 100 users with EndNote 20. This is ideal for large group projects, research teams, and large systematic review teams.

Additional upgrades were made in the back end to help with the recovery of corrupt library files. EndNote libraries can become corrupt, and EndNote recommends not saving libraries in cloud or network storage which are notorious for corrupting library files. However, there are new tools to help users recover lost data by synching with an online account. In addition, if a user makes a change to a record but then needs to go back to the original record, there is now a “compare versions” option in the “Edit” tab of each record.

For those writing in groups, End-Note 21 is now compatible with Google Docs by using the “Cite While You Write” extension that connects to a user's online EndNote account. To add the extension, click on the Extension tab at the top of the Google Docs, go to Add Ons, and install the “EndNote Cite While You Write” extension. Unlike Word, where an EndNote tab appears in the top navigation bar, in Google Docs a user goes to Extensions, then to “EndNote Cite While You Write” then clicks on Open. “Cite While You Write” opens in the left-hand sidebar. The user interface is intuitive and for users who are already familiar using EndNote with Word, there is no learning curve to use EndNote with Google Docs. When testing, there was a noticeable lag in the time from inserting the citation and the reference appearing, and when moving sentences, the citations and references did not update. In addition, when the document was downloaded into Word and additional citations were added, they appeared in a separate reference list at the bottom of the document. However, the combination of Google Docs with EndNote is still ideal for group projects, allowing all writers the ability to add citations to manuscripts easily.

## RETRACTION WATCH

EndNote 21 is integrated with Retraction Watch. Databases, like Web of Science and PubMed, support exporting large numbers of records to EndNote at a single time. For users who download large numbers of records, the integration with Retraction Watch is beneficial. Within a few minutes of importing a record EndNote automatically detects if the article is included in the Retraction Watch website. When one or more retracted articles are found EndNote adds a Retractions folder in the left-hand bar. The article also shows a retracted publication notice. To further help users avoid citing retracted articles, EndNote displays a warning when adding a retracted citation to a Word document. This is especially useful to users with long-term projects, as End-Note scans the library when opened, helping to ensure newly retracted articles are not cited. To enable the Retraction Watch features users must have a free EndNote online account.

## ENDNOTE 21 EXCLUSIVE ONLINE INTERFACE

With EndNote 21 comes an exclusive offer for an upgraded online EndNote user interface. For three years users will have unlimited storage space for records and attachments. It is not clear what happens at the end of the three-year period. The new user interface is similar to the EndNote desktop version. For users who are familiar with EndNote Web, the user interface has eliminated the top navigation for Collect, Organize, Format, Match, Options, and Downloads. According to a support chat with Clarivate, the development team is still working on the full functionality of the new online product, and it was recommended to continue using the older version at this time. Clarivate was unable to provide a timeline for when the product would be fully functioning.

## ENDNOTE FOR SYSTEMATIC REVIEWS

EndNote 21, combined with EndNote Web, can be used for systematic reviews. Librarians who support systematic reviews can run searches in multiple databases and export all results to EndNote. The results can be deduplicated in the desktop version or in the older online version, and the library easily shared with the systematic review team. As systematic reviews can take 12-24 months, the automatic updating of retracted articles when the library is opened will help the team avoid retracted articles in their reviews. EndNote is also compatible with several Systematic Review tools including, JBI SUMARI, Covidence, DistillerSR, and Raayan. It is not necessary for everyone on the team to have EndNote 21 as it is still compatible with older versions and the online version.

## TRAINING

Trainings for Endnote 21 include video tutorials, self-guided learning, reference guides, live trainings, and recorded webinars [1]. Clarivate uses LibGuides, and librarians may find the familiar guides easy to navigate. The tab “For Trainers” includes four documents, misleadingly labeled as “class handouts”. The “class handouts” are one-page documents with a session goal and learning objectives that will benefit librarians who are creating teaching sessions on EndNote but are not documents that would be distributed to a class. The handouts range from essential to expert and could be a great starting point for librarians who are new to teaching EndNote. EndNote does provide user guides that would be more beneficial as class handouts. In addition, the new training videos once again include audio, and closed captioning for accessibility. Available on YouTube, “How to use EndNote 21 in Seven Minutes,” is available for Windows and Mac users in English. Live trainings are available upon request. As of July 18, 2023, there were no training materials for the new EndNote online.

## PRICING

For those who have EndNote 20 or earlier, a single license upgrade costs $124.95, and is a one-time purchase. For those new to EndNote a full license is a one-time purchase of $274.95. College and university students should first check with their library to see if EndNote is provided before purchasing the full license with the student discount of $149.95. For EndNote 20 users it may not be worth the cost to upgrade with this release.

## CONCLUSION

For experienced users upgrading, End-Note 21 has a small learning curve to take advantage of the new tags, otherwise, a user's workflow remains the same. Those new to EndNote 21 should take advantage of their tutorials to quickly begin using this powerful program and start saving time and the hassle of managing and citing references.
